# Quantifying the Unknowns of Plaque Morphology: The Role of Topological Uncertainty in Coronary Artery Disease

**DOI:** 10.1016/j.mcpdig.2025.100217

**Published:** 2025-03-28

**Authors:** Yashbir Singh, Quincy A. Hathaway, Karthik Dinakar, Leslee J. Shaw, Bradley Erickson, Francisco Lopez-Jimenez, Deepak L. Bhatt

**Affiliations:** a Department of Radiology, Mayo Clinic, Rochester, MN; b Department of Radiology, University of Pennsylvania, Philadelphia, PA; c Mount Sinai Fuster Heart Hospital, Icahn School of Medicine, New York, NY; d Massachusetts Institute of Technology, Cambridge, MA; e Fujita Health University, Toyoake, Aichi, Japan; f Chiba Institute of Technology, Chiba, Japan; g Blavatnik Family Research Institute, Icahn School of Medicine at Mount Sinai, New York, NY; h Department of Cardiovascular Medicine, Mayo Clinic, Rochester, MN

## Abstract

This article aimed to explore topological uncertainty in medical imaging, particularly in assessing coronary artery calcification using artificial intelligence (AI). Topological uncertainty refers to ambiguities in spatial and structural characteristics of medical features, which can impact the interpretation of coronary plaques. The article discusses the challenges of integrating AI with topological considerations and the need for specialized methodologies beyond traditional performance metrics. It highlights advancements in quantifying topological uncertainty, including the use of persistent homology and topological data analysis techniques. The importance of standardization in methodologies and ethical considerations in AI deployment are emphasized. It also outlines various types of uncertainty in topological frameworks for coronary plaques, categorizing them as quantifiable and controllable or quantifiable and not controllable. Future directions include developing AI algorithms that incorporate topological insights, establishing standardized protocols, and exploring ethical implications to revolutionize cardiovascular care through personalized treatment plans guided by sophisticated topological analysis. Recognizing and quantifying topological uncertainty in medical imaging as AI emerges is critical. Exploring topological uncertainty in coronary artery disease will revolutionize cardiovascular care, promising enhanced precision and personalization in diagnostics and treatment for millions affected by cardiovascular diseases.

### Setting

Coronary artery calcification (CAC) is a crucial biomarker for cardiovascular disease risk^1^, ^2^, ^3^ predisposing patients to myocardial infarction and stroke. However, accurate assessment of CAC, as well as noncalcified plaques, can be complicated by uncertainty (ie, variations in acquisition, postprocessing, and viewing images). In medical imaging, uncertainty is a pivotal yet enigmatic concept, especially within artificial intelligence (AI). This article aimed to explore uncertainty in topology, unraveling its impact on the interpretation and analysis of coronary plaques through the lens of AI-enhanced medical imaging. Topological uncertainty^4^ refers to ambiguity surrounding the spatial, structural, and shape-related characteristics of medical features in imaging data, stemming from the inherent variability in human anatomy and disease, limitations of imaging technologies, and diverse disease manifestations. In the realm of coronary plaque detection, this form of uncertainty can significantly alter the perceived extent and severity of calcification, potentially leading to misdiagnoses, inadequate treatment plans, and misguided research endeavors. These challenges are particularly relevant in the current era of medical imaging, where AI is increasingly deployed for analysis.

The transformative role of AI in medical imaging offers unprecedented precision and efficiency in analyzing complex data.^5^^,^^6^ However, integrating AI introduces a spectrum of uncertainties, with topological uncertainty particularly challenging.^7^ It demands a nuanced understanding of underlying topological features—for example, connectedness, compactness, and the presence of voids—to accurately interpret medical images.

Addressing topological uncertainty in coronary artery disease (CAD) necessitates innovation beyond traditional model performance metrics like accuracy, precision, and recall. These fail to capture the intricacies of topological errors, highlighting the need for specialized methodologies to quantify and manage topological uncertainty. Emerging topological data analysis (TDA) techniques promise a comprehensive framework for analyzing data’s shape and connectivity.^6^

The implications of topological uncertainty^4^ extend beyond diagnostic accuracy. Misjudging the boundaries and composition of coronary plaques in treatment planning could lead to misguided intervention, adversely affecting patient outcomes. The fusion of insights from mathematics, computer science, and medical science is pivotal in developing comprehensive solutions that address the challenges posed by topological uncertainty in CAD.^8^

### Approach

#### Advancements in Quantifying Topological Uncertainty

The quest to quantify topological uncertainty has significantly advanced TDA, particularly in developing persistent homology.^4^^,^^6^ This method has been instrumental in identifying and quantifying topological features across multiple scales, revealing the inherent data structures that traditional analysis might overlook.

To validate these theoretical advances in practical settings, recent multicenter validation studies of automated coronary calcium scoring systems have found the practical application of these principles, showing robust performance across diverse clinical settings while effectively managing varying degrees of image quality and anatomical uncertainty.^9^ These advances in automation, combined with rigorous validation across multiple centers, underscore the feasibility of implementing sophisticated topological analysis in routine clinical practice.^10^

These validation efforts reported clinical feasibility and reproducibility across diverse health care settings, with performance metrics showing consistent calcium quantification despite variations in scanner technology and patient populations. A key example from clinical practice involves a 65-year-old male patient whose coronary calcium score interpretation varied by 15% across different analysis methods, highlighting how topological uncertainty directly impacts risk stratification and potentially treatment decisions. Such real-world examples underscore the practical significance of addressing topological uncertainty in routine cardiovascular care.

Applying persistent homology to plaque formation allows researchers to understand geometric and topological nuances better,^9^ leading to more precise cardiovascular risk assessments. Types of uncertainty in topological frameworks for coronary plaques include (Figure) the following:

1. Quantifiable and controllable
  a. Measurement: How good is our equipment?
  b. Model: How do we define coronary plaques?
  c. Parameter: How homogenous/heterogeneous is the region of interest?
  d. Statistical: How big is the region of interest?
  e. Environmental: Can the patient follow imaging protocol?
  f. Human: Has the patient had previous thoracic surgery? (Few patients would be in this category with previous cardiothoracic surgery. What about other structural issues: myocardial bridges, tortuous or small vessels? Or valvular calcification?)
2. Quantifiable and not controllable
  a. Epistemic: Unknown characteristics of coronary plaques. One unexplored approach is to use CAC to understand the burden of noncalcified plaque. Can this be done with shape/location, and so on, so not really unknown but not visualized characteristics of coronary plaques?
  b. Aleatoric: inherent randomness of acquired data.

**Figure:**
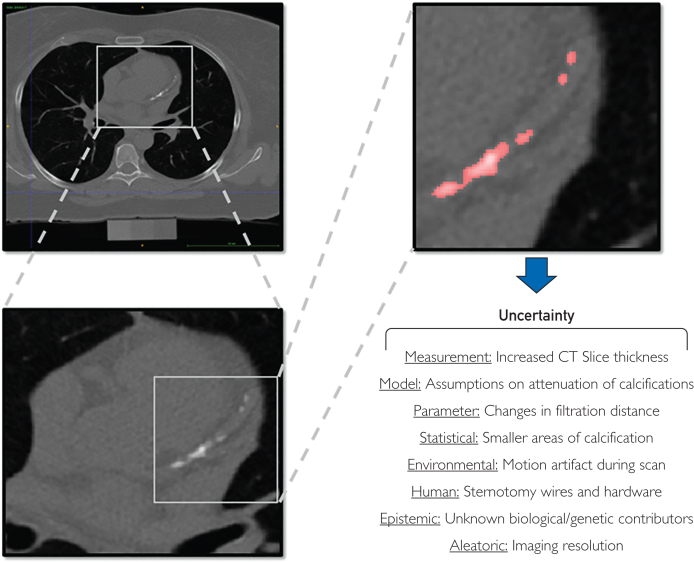
Noncontrast computed tomography (CT) coronary artery calcium scan. How can topological data analysis assess coronary artery calcium? What types of uncertainty should be considered when evaluating the model output?

### Application

#### Integrating AI With Topological Insights

Measuring uncertainty in AI is a long-standing core branch of computer science. Integrating AI with topological features represents a frontier in tackling model inference challenges posed by topological uncertainty. AI algorithms, especially those using deep learning, have shown remarkable proficiency in identifying complex data set patterns and features. However, by incorporating topological considerations, such as the presence of loops or voids indicative of plaque patterns, these algorithms could achieve higher accuracy and reliability. This approach can improve diagnostic precision and enhance the model’s ability to predict clinical outcomes, potentially guiding therapeutic decision making.^11^, ^12^, ^13^ The format, structure, and manner in which the model’s uncertainty is communicated to the physician are an important area for exploration. These explorations extend beyond basic calcium quantification. Beyond traditional calcium scoring, advanced AI techniques have established capability in analyzing complex cardiac structures and surrounding tissues, with Commandeur et al^14^ showing how deep learning can reliably quantify cardiac-adjacent features while accounting for anatomical variations.^14^

Specifically, convolutional neural networks enhanced with topological feature extractors have shown promise in capturing both local and global structural characteristics of coronary plaques. Compared with conventional calcium scoring methods that rely on Agatston units, these TDA-integrated approaches provide superior characterization of plaque morphology and distribution, potentially offering more refined risk stratification. Recent frameworks using U-Net architectures augmented with persistent homology features have reported up to 12% improvement in detection accuracy compared with standard deep learning models when evaluating complex, mixed-composition plaques.

##### The Role of Standardization

Standardization in the methodologies used to quantify and report topological features is critical for addressing challenges of topological uncertainty in CAD. Establishing standardized protocols for TDA in medical imaging ensures reproducible and comparable findings across studies, enhancing knowledge and facilitating clinical application. Specifically, standardizing the pipeline for processing medical images, from acquisition to analysis, can significantly reduce variability. For example, persistent homology, a method in TDA, offers a rigorous framework for quantifying shapes and features within data, which can help identify patterns of calcium deposition across patient cohorts. Another real example is Mapper,^15^ an algorithm with simplified representation of high-dimensional data, which can be standardized to visualize and analyze vascular structures. By integrating these methods into a cohesive platform, researchers and clinicians can approach the complex variability of CAD with a more unified and precise lens. This not only aids in the accurate assessment and prognosis of cardiovascular diseases but also paves the way for personalized treatment plans.^16^

Despite these promising standardization efforts, implementation faces notable challenges including variability in imaging protocols across institutions, computational resource requirements for complex topological analyses, and current regulatory frameworks that may not fully accommodate novel TDA-based biomarkers. Additionally, integration with existing clinical workflows requires balancing sophisticated analysis with practical time constraints in busy clinical environments. Nevertheless, incremental adoption focused on high-risk patient cohorts could provide a pathway for broader implementation.

These findings in AI integration and standardization highlight both the progress made and the challenges ahead in addressing topological uncertainty in CAD. The following discussion explores the broader implications and future directions of these developments.

### Findings

Recent advances in cardiovascular imaging have established the transformative potential of AI in risk stratification for CAD.^17^ This evolution in risk assessment methodology has shown how AI can systematically analyze imaging features while accounting for various sources of uncertainty, highlighting its potential to enhance clinical decision making through more comprehensive risk evaluation. The integration of AI-driven risk stratification tools represents a significant advancement toward more reliable and personalized patient assessment, particularly when considering the complex interplay between imaging features and clinical outcomes.^18^ However, this advancement brings important ethical considerations that must be carefully addressed.

### Ethical Considerations

The potential for topological uncertainty to impact patient outcomes necessitates a cautious approach to AI deployment in medical imaging.^17^^,^^19^ Transparency in the algorithms’ workings, awareness of their limitations, and continuous bias monitoring are essential to ensuring that these advanced tools serve patients’ best interests.^20^^,^^21^

### Discussion

Quantifying topological uncertainty for CAD could focus on advancing topological data analysis techniques to enhance cardiovascular risk assessments.^22^ This may involve developing AI algorithms that incorporate topological insights for improved diagnostic accuracy and establishing standardized protocols for analyzing and reporting topological features in medical imaging. Researchers may integrate persistent homology, uncertainty, and other TDA methods into clinical decision making processes.^22^ Likely, there will be increased emphasis on investigating the ethical implications of AI deployment in medical imaging, with a focus on transparency and bias mitigation. Ultimately, these efforts aim to further personalize treatment plans with sophisticated topological analysis of plaque patterns, revolutionizing cardiovascular care. Recognizing and quantifying topological uncertainty in medical imaging and AI emerges is critical. Looking to the future, exploring topological uncertainty in CAD will revolutionize cardiovascular care, promising enhanced precision and personalization in diagnostics and treatment for millions affected by cardiovascular diseases.

## Potential Competing Interests

Dr Bhatt is in the advisory board of Angiowave, Bayer, Boehringer Ingelheim, CellProthera, Cereno Scientific, E-Star Biotech, High Enroll, Janssen, Level Ex, McKinsey, Medscape Cardiology, Merck, NirvaMed, Novo Nordisk, Stasys; and Tourmaline Bio; is one of the board of directors in American Heart Association New York City; holds stock or stock options in Angiowave, Bristol-Myers Squibb, DRS.LINQ, and High Enroll; is a consultant for Broadview Ventures, Corcept Therapeutics, GlaxoSmithKline, Hims, SFJ, Summa Therapeutics, and Youngene; is in the data monitoring committee of Acesion Pharma, Assistance Publique-Hôpitaux de Paris, Baim Institute for Clinical Research (formerly Harvard Clinical Research Institute, for the PORTICO trial, funded by St. Jude Medical, now Abbott), Boston Scientific (Chair, PEITHO trial), Cleveland Clinic, Contego Medical (Chair, PERFORMANCE 2), Duke Clinical Research Institute, Mayo Clinic, Mount Sinai School of Medicine (for the ENVISAGE trial, funded by Daiichi Sankyo; for the ABILITY-DM trial, funded by Concept Medical; for ALLAY-HF, funded by Alleviant Medical), Novartis, Population Health Research Institute; and Rutgers University (for the NIH-funded MINT Trial); reports honoraria from American College of Cardiology (Senior Associate Editor, Clinical Trials and News, ACC.org; Chair, ACC Accreditation Oversight Committee), Arnold and Porter law firm (work related to Sanofi/Bristol-Myers Squibb clopidogrel litigation), Baim Institute for Clinical Research (formerly Harvard Clinical Research Institute; AEGIS-II executive committee funded by CSL Behring), Belvoir Publications (Editor-in-Chief, Harvard Heart Letter), Canadian Medical and Surgical Knowledge Translation Research Group (clinical trial steering committees), CSL Behring (AHA lecture), Cowen and Company, Duke Clinical Research Institute (clinical trial steering committees, including for the PRONOUNCE trial, funded by Ferring Pharmaceuticals), HMP Global (Editor-in-Chief, *Journal of Invasive Cardiology*), *Journal of the American College of Cardiology* (Guest Editor; Associate Editor), Level Ex, Medtelligence/ReachMD (CME steering committees), MJH Life Sciences, Oakstone CME (Course Director, Comprehensive Review of Interventional Cardiology), Piper Sandler, Population Health Research Institute (for the COMPASS operations committee, publications committee, steering committee, and USA national co-leader, funded by Bayer), and WebMD (CME steering committees), Wiley (steering committee); and other relationships as follows: *Clinical Cardiology* (Deputy Editor); patent: Sotagliflozin (named on a patent for sotagliflozin assigned to Brigham and Women’s Hospital who assigned to Lexicon; neither I nor Brigham and Women’s Hospital receive any income from this patent); research funding: Abbott, Acesion Pharma, Afimmune, Aker Biomarine, Alnylam, Amarin, Amgen, AstraZeneca, Bayer, Beren, Boehringer Ingelheim, Boston Scientific, Bristol-Myers Squibb, Cardax, CellProthera, Cereno Scientific, Chiesi, CinCor, Cleerly, CSL Behring, Faraday Pharmaceuticals, Ferring Pharmaceuticals, Fractyl, Garmin, HLS Therapeutics, Idorsia, Ironwood, Ischemix, Janssen, Javelin, Lexicon, Lilly, Medtronic, Merck, Moderna, MyoKardia, NirvaMed, Novartis, Novo Nordisk, Otsuka, Owkin, Pfizer, PhaseBio, PLx Pharma, Recardio, Regeneron, Reid Hoffman Foundation, Roche, Sanofi, Stasys, Synaptic, The Medicines Company, Youngene, and 89Bio; and royalties from Elsevier (Editor, Braunwald’s Heart Disease); and is a site co-investigator for Cleerly. All other authors have no relevant disclosures. Given their role as Editor-in-Chief and Editorial Board Member, Dr Francisco Lopez-Jimenez and Dr Bradley Erickson were not involved in the peer-review of this article and have no access to information regarding its peer-review. Full responsibility for the editorial process for this article was delegated to an unaffiliated Editor.

## References

[1] Budoff M.J., Hokanson J.E., Nasir K. (2010). Progression of coronary artery calcium predicts all-cause mortality. JACC Cardiovasc Imaging.

[2] Budoff M.J., Young R., Burke G. (2018). Ten-year association of coronary artery calcium with atherosclerotic cardiovascular disease (ASCVD) events: the multi-ethnic study of atherosclerosis (MESA). Eur Heart J.

[3] Kakadiaris I.A., Vrigkas M., Yen A.A., Kuznetsova T., Budoff M., Naghavi M. (2018). Machine learning outperforms ACC/AHA CVD risk calculator in MESA. J Am Heart Assoc.

[4] Gupta S., Zhang Y., Hu X., Prasanna P., Chen C. (2024). Topology-aware uncertainty for image segmentation. Adv Neural Inf Process Syst.

[5] Nurmohamed N.S., van Rosendael A.R., Danad I. (2024). Atherosclerosis evaluation and cardiovascular risk estimation using coronary computed tomography angiography. Eur Heart J.

[6] Singh Y., Farrelly C.M., Hathaway Q.A. (2023). Topological data analysis in medical imaging: current state of the art. Insights Imaging.

[7] Guo F., Ng M., Kuling G., Wright G. (2022). Cardiac MRI segmentation with sparse annotations: ensembling deep learning uncertainty and shape priors. Med Image Anal.

[8] Oren O., Blankstein R., Bhatt D.L. (2020). Incidental imaging findings in clinical trials. JAMA.

[9] Singh Y., Farrelly C., Hathaway Q.A. (2023). The role of geometry in convolutional neural networks for medical imaging. Mayo Clin Proc Digit Health.

[10] Eng D., Chute C., Khandwala N. (2021). Automated coronary calcium scoring using deep learning with multicenter external validation. NPJ Digit Med.

[11] Zia A., Khamis A., Nichols J. (2024). Topological deep learning: a review of an emerging paradigm. Artif Intell Rev.

[12] Srinivasan G., Hyman J.D., Osthus D.A. (2018). Quantifying topological uncertainty in fractured systems using graph theory and machine learning. Sci Rep.

[13] Faghani S., Moassefi M., Rouzrokh P. (2023). Quantifying uncertainty in deep learning of radiologic images. Radiology.

[14] Commandeur F., Goeller M., Betancur J. (2018). Deep learning for quantification of epicardial and thoracic adipose tissue from non-contrast CT. IEEE Trans Med Imaging.

[15] Singh G., Mémoli F., Carlsson G.E. (2007). Topological methods for the analysis of high dimensional data sets and 3D object recognition. PBG@Eurographics.

[16] Freeman A.M., Raman S.V., Aggarwal M. (2023). Integrating coronary atherosclerosis burden and progression with coronary artery disease risk factors to guide therapeutic decision making. Am J Med.

[17] Tat E., Bhatt D.L., Rabbat M.G. (2020). Addressing bias: artificial intelligence in cardiovascular medicine. Lancet Digit Health.

[18] Lin A., Kolossváry M., Motwani M. (2021). Artificial intelligence in cardiovascular imaging for risk stratification in coronary artery disease. Radiol Cardiothorac Imaging.

[19] Oren O., Gersh B.J., Bhatt D.L. (2020). Artificial intelligence in medical imaging: switching from radiographic pathological data to clinically meaningful endpoints. Lancet Digit Health.

[20] Akinci D'Antonoli T. (2020). Ethical considerations for artificial intelligence: an overview of the current radiology landscape. Diagn Interv Radiol.

[21] Oren O., Gersh B.J., Bhatt D.L. (2021). Improving communication of incidental imaging findings: transforming uncertainty into opportunity. Mayo Clin Proc.

[22] Singh Y., Hathaway Q.A., Farrelly C. (2025). Topological data analysis in the assessment of coronary atherosclerosis: a comprehensive narrative review. Mayo Clin Proc Digit Health.

